# Strategic Considerations for Video Consultation Implementation in General Practice: Qualitative Study of Organizational Interest-Holders’ Perspectives

**DOI:** 10.2196/106707

**Published:** 2026-10-19

**Authors:** Ulrik Bak Kirk, Ditte Bonde Stanek, Mette Amalie Nebsbjerg, Flemming Bro, Linda Huibers, Adrian Rohrbasser, Anna Mygind, Viola Burau, Ida Hestbjerg

**Affiliations:** 1 Department of Public Health Faculty of Medicine Aarhus University Aarhus, Central Denmark Region Denmark; 2 Research Unit for General Practice Aarhus, Central Denmark Region Denmark; 3 Department for Primary Care and Public Health, Global Digital Health Unit Imperial College London London, England United Kingdom; 4 Institute of Primary Health Care (BIHAM) Faculty of Medicine University of Bern Bern, Bern Switzerland

**Keywords:** digital health, digital transformation, general practice, implementation, interest-holder, organizational perspectives, primary care, qualitative research, telemedicine, video consultation

## Abstract

**Background:**

Video consultations have become an established component of general practice and are increasingly promoted to improve access, efficiency, and health care sustainability. While previous research has primarily explored the perspectives of individual patients and health care professionals, less is known about how organizations involved in strategic video consultation implementation consider their benefits, challenges, and future role within health care systems.

**Objective:**

This study aims to explore the experiences of organizational interest-holders involved in implementing video consultations in Danish general practice, focusing on the perceived strengths, weaknesses, opportunities, and threats related to their use.

**Methods:**

A qualitative interview study was conducted with 17 representatives from 13 organizations involved in the development, support, regulation, governance, or strategic implementation of video consultations in Denmark. Purposive and snowball sampling identified participants from 4 different organizational groups: patient organizations, professional interest organizations, technology and digital health organizations, and public health authorities. Semistructured interviews were conducted between October and December 2023. Two researchers analyzed the transcripts using a 5-stage deductive-inductive framework analysis. Interest-holder theory defined whose role-informed perspectives were compared; the Strengths, Weaknesses, Opportunities, and Threats (SWOT) analysis framework structured the initial descriptive charting; and iterative comparisons across SWOT categories, organizational groups, and 5 analytical levels identified patterns of convergence, divergence, and tension.

**Results:**

Four overarching tensions, competing values, and dilemmas emerged across the organizational interest-holder groups: (1) improved access versus widening inequity, (2) efficiency versus relational and clinical quality and safety, (3) digital transformation versus implementation uncertainty, and (4) strategic governance versus target-driven digitalization. Participants recognized that video consultations may improve accessibility, convenience, and coordination of care while supporting broader digital transformation. However, concerns were raised regarding digital exclusion, limitations in relational and clinical assessment, uncertainty about appropriate use, cross-sector implementation challenges, and governance approaches that prioritize use targets over clinical value, quality, and patient safety.

**Conclusions:**

Exploring the perspectives of organizational interest-holders suggested that implementing video consultations in general practice is not simply a matter of increasing technology uptake. Rather, it requires balancing competing priorities related to access and equity, efficiency and quality and safety, digital transformation and implementation uncertainty, and governance and professional autonomy. Sustainable implementation should promote equitable access, context-sensitive clinical use, interoperable digital infrastructure, and good governance. This should be based on research that assesses when video consultations add value, for whom, and according to which definitions of quality and safety, rather than prioritizing uptake alone.

## Introduction

### Background

Digital health technologies have become an essential component in contemporary health care systems, transforming how health care services are accessed, delivered, and organized [1,2]. Although digitalization has been a strategic priority in many countries for decades [3-7], the COVID-19 pandemic substantially accelerated the adoption of remote care models, including video consultations, as health care systems sought to maintain access to care while reducing unnecessary physical contact and infection risks [8-11]. Across many health care systems, video consultations have subsequently been promoted as a means of improving accessibility, enhancing convenience for patients and professionals, and supporting more efficient use of health care resources [12,13].

Many health care systems rely on general practice as a cornerstone of care delivery, providing first-contact access, continuity, coordination, and gatekeeping functions [14,15]. Consequently, the implementation of video consultations in general practice has attracted considerable policy attention. Denmark provides a particularly relevant case because it is among the world’s most digitally advanced health care systems, supported by a secure national digital infrastructure and long-standing governmental strategies for health care digitalization [16-19]. Well before the COVID-19 pandemic, Danish digital health strategies emphasized telemedicine and video consultations as mechanisms for improving access to care irrespective of geography and for increasing efficiency within health care services [20,21]. The pandemic accelerated these developments but did not initiate them.

Despite sustained policy interest and substantial investments, uncertainty remains regarding the benefits, limitations, implementation barriers, unintended consequences, and long-term role of video consultations in general practice [22-25]. These uncertainties are not merely technical or logistical; they also concern how recurring tensions, competing values, and dilemmas are balanced in and affect implementation.

### Research Gap and Objectives

Existing research has predominantly focused on the individual perspectives of patients and health care professionals [26-28]. Comparatively less attention has been given to other interest-holders involved in implementation, including organizations responsible for developing digital infrastructure, delivering technological solutions, regulating health care services, shaping policy, and representing collective professional or patient interests. Understanding these organizational perspectives is important because successful implementation depends not only on technological capability but also on the interactions between multiple organizations whose interests, responsibilities, and priorities influence the adoption, governance, and sustainability of digital health innovations.

To explore these organizational perspectives systematically, this study draws on a SWOT (Strengths, Weaknesses, Opportunities, and Threats) analysis framework, which is widely used to identify internal and external factors that may facilitate or hinder implementation processes [29-31]. Rather than treating implementation as a linear process of technological adoption, this framework enables examination of how perceived benefits and risks may coexist and differ across interest-holder groups.

Building on the limited evidence regarding nonclinical and organizational interest-holder perspectives, this study aims to explore the experiences of organizational interest-holders involved in implementing video consultations in Danish general practice, focusing on the perceived strengths, weaknesses, opportunities, and threats related to their use.

## Methods

### Theoretical Framework

Interest-holder engagement has become increasingly important in health research, policy development, and implementation science. Interest-holder theory conceptualizes “interest-holders” as groups with legitimate interests arising from being responsible for, affected by, or able to influence health-related decisions and interventions [32]. Interest-holders may represent patient interests, professional interests, commercial interests, or public responsibilities and therefore contribute different forms of knowledge, experience, and accountability. Evidence suggests that engaging relevant interest-holders may improve the relevance, applicability, and implementation of health research, particularly in studies intended to inform health care policy, service delivery, and guideline development [33,34].

By ensuring that research reflects real-world priorities and realities, such engagement may strengthen the practical usefulness and relevance of research findings [35]. Involving those with legitimate interests promotes transparency, accountability, and fairness and aligns with ethical principles such as respect for persons and justice, as articulated in the Belmont Report and the Council for International Organizations of Medical Sciences (CIOMS) guidelines [36,37].

In the context of video consultations in general practice, relevant interest-holders extend beyond patients and health care professionals. They also include organizations responsible for developing digital infrastructure, providing technological solutions, regulating health care services, shaping digital health policy, representing professional interests, and advocating for patients [22,29,31]. These organizations influence both the implementation and long-term sustainability of video consultations.

Digital transformation in health care therefore represents not only a technological change process, but also an organizational, regulatory, and relational one [38,39]. Different interest-holder groups may experience digital transformation differently because their responsibilities, interests, mandates, and organizational contexts vary. Understanding these perspectives requires analytical approaches capable of capturing both normative concerns and practical implementation challenges.

To address this complexity, a SWOT analysis was adopted as the analytical framework for this study. A SWOT analysis enables the distinction between internal organizational factors (strengths and weaknesses) and external contextual conditions (opportunities and threats) that may facilitate or constrain implementation [40]. The approach has been widely applied in health services research to examine digital health policies, implementation strategies, and broader health care system challenges [29,30,41-44].

Combining interest-holder theory with a SWOT analysis provides a framework for examining how the interests and experiences of different groups intersect with organizational and system-level conditions influencing the implementation of video consultations in general practice. This combined approach is particularly relevant where the same feature of an intervention may be interpreted differently across groups, for example as a strength from one organizational perspective but as a weakness or threat from another.

### Study Design

To address the study aim, we adopted a qualitative design based on semistructured interviews with representatives from organizations identified as relevant interest-holders in the implementation of video consultations in Danish general practice [45,46]. The study’s design, conduct, and reporting were guided by the COREQ (Consolidated Criteria for Reporting Qualitative Research) checklist to enhance transparency, reflexivity, and methodological rigor [47].

### Setting

General practice in Denmark is publicly funded, with services provided free of charge to registered patients. During daytime opening hours (8 AM-4 PM), general practitioners and general practice staff provide care through face-to-face, telephone, email, home, and video consultations. General practice remuneration combines capitation with fee-for-service payments, making accurate coding of all services financially important [48].

Although a reimbursement code for video consultations existed before the COVID-19 pandemic, their uptake remained minimal until their nationwide emergency rollout in April 2020, which facilitated broader implementation in Danish general practice [10,24]. Video consultations became a permanent consultation modality in January 2022, and since late 2024 all general practices in Denmark have been required to offer them [25]. To support adoption, video consultations were initially reimbursed at a slightly higher rate than face-to-face consultations, although reimbursement was equalized in January 2025 [24,25].

### Study Participants

Participants were recruited from organizations involved in the implementation, governance, regulation, support, or strategic development of video consultations within the Danish health care system. Consistent with the study’s interest-holder perspective, four categories of organizations were purposively targeted: (1) patient organizations, (2) professional interest organizations, (3) technology and digital health organizations, and (4) public health authorities, as shown in Table 1.

A purposive sampling strategy was applied, informed by national policy documents [20,21] and previous research on the implementation of video consultations across multiple levels of the health care system [22]. To ensure that key organizational perspectives were represented, organizations were selected to cover 4 distinct functions within the implementation of digital health and video consultations: patient representation, professional representation, technology or infrastructure provision, and public governance or regulation. Sampling sought coverage of distinct organizational roles in video consultation implementation rather than numerical balance between the 4 interest-holder groups. Snowball sampling was subsequently used during interviews to identify additional relevant organizations. Recruitment was conducted through email invitations sent to representatives with relevant expertise and responsibilities within each organization. In total, 18 organizations were invited to participate, of which 13 agreed. Some interviews involved 2 organizational representatives, resulting in a total of 17 participants. The distribution across the 4 interest-holder groups is presented in Table 2.

Participants held senior or specialist positions within their respective organizations and contributed perspectives grounded in governance, policy development, implementation, advocacy, or technological provision related to video consultations. Their tenure in current roles ranged from less than 12 months to 14 years, reflecting both recent and long-standing engagement with digital health initiatives.

**Table 1:** Definitions of interest-holder groups.

Category	Interest-holder group	Definition
1	Patient organizations	Organizations representing patient and citizen interests in health care services
2	Professional interest organizations	Organizations representing health care professionals and their professional interests regarding health care delivery
3	Technology and digital health organizations	Organizations involved in developing, delivering, supporting, or coordinating digital health technologies, infrastructures, and services
4	Public health authorities	Public organizations responsible for health care governance, regulation, policy development, strategic planning, or oversight of digital health services

**Table 2:** Number of interviews and participants across interest-holder organizations.

Interviews (n=13) and participants (n=17)	Interest-holder organizations			
	Patient (n=3)	Professional (n=2)	Technology and digital health (n=4)	Public health (n=4)
1 (P1)			✓	
2 (P2 and P3)				✓
3 (P4)				✓
4 (P5 and P6)				✓
5 (P7)	✓			
6 (P8)	✓			
7 (P9)			✓	
8 (P10)			✓	
9 (P11)	✓			
10 (P12 and P13)		✓		
11 (P14)			✓	
12 (P15)		✓		
13 (P16 and P17)				✓

### Data Collection

Semistructured, in-depth interviews were conducted online via Zoom (Zoom Communications, Inc) or Microsoft Teams between October and December 2023 and lasted 34-50 minutes. The topic guide, including questions on perceived strengths, weaknesses, opportunities, and threats, was informed by previous research [20,30]. The topic guide is provided in Multimedia Appendix 1. All interviews were recorded and transcribed verbatim. Transcripts were stored securely on protected servers. All interviews were conducted in Danish by 1 or 2 researchers who had no prior relationship with the participants, thereby minimizing the potential for bias or preconceived assumptions.

Participants were invited to reflect primarily in their capacity as representatives of organizational interest-holders; however, some indicated when the views expressed reflected personal perspectives rather than organizational positions. Accordingly, “organizational perspectives” refers in this study to representatives’ role-informed accounts rather than formally authorized positions of their organizations. Parts of this study, specifically participant recruitment, data collection, and initial analysis, were conducted in collaboration with the cross-sectoral innovation project centered on patient-generated data Phase V [49].

### Data Analysis

The interview transcripts were independently reviewed and analyzed by 2 researchers using framework analysis following five stages: (1) familiarization with the data, (2) development of an analytical framework, (3) indexing, (4) charting, and (5) mapping and interpretation [30]. Analysis combined deductive and inductive approaches [50,51].

Deductively, data were initially organized according to the study aim, topic guide, SWOT analysis, and 5 analytical framework levels (patients, health care organization units, health care systems, legislation and regulation, and technology and infrastructure) [30]. Inductively, researchers remained attentive to themes and patterns emerging from the data that were not anticipated a priori. As analysis progressed, no substantially new themes emerged, and we interpreted this as sufficient thematic coverage for the study aim.

Coding and interpretation were conducted iteratively, with consensus discussions used to refine the analytical framework and resolve discrepancies. Interest-holder theory defined whose role-informed perspectives were compared, SWOT analysis structured the initial descriptive charting, and framework analysis provided the iterative procedure for comparing data across interest-holder groups and analytical levels. The initial analysis generated the strengths, weaknesses, opportunities, and threats summarized in Multimedia Appendix 2.

The subsequent interpretive analysis examined patterns of convergence, divergence, tension, and overlap across the SWOT categories, 4 interest-holder groups, and 5 analytical framework levels. For example, the findings summarized in Table S1 in Multimedia Appendix 2 showed that improved accessibility and flexibility, reduced travel and time burdens, lower thresholds for contacting general practice, and the potential inclusion of underserved or mobility-limited groups were charted as patient-level strengths or opportunities. At the same time, uneven digital competence, misalignment with patient preferences, widening digital inequities, potential safety concerns for vulnerable patients, and the risk of resources shifting away from those requiring nondigital alternatives were charted as weaknesses or threats. Comparing these findings across interest-holder groups demonstrated that the same development could improve access for some patients while creating or reinforcing barriers for others. This interpretive insight generated the higher-order theme “improved access versus widening inequity.” Applying the same comparative process across the remaining categories and analytical levels resulted in 4 higher-order themes reflecting recurring tensions, competing values, and dilemmas associated with implementing video consultations in general practice. Multimedia Appendix 2 presents both the initial SWOT analysis and the progression from the descriptive findings to these higher-order themes.

### Reflexivity

The multidisciplinary research team comprised researchers with expertise in general practice and digital public health and extensive experience in qualitative research. Several members of the team have previously conducted research on video consultations and digital health implementation. Throughout data collection and analysis, researchers actively discussed how their professional backgrounds, prior knowledge, and potential assumptions regarding digital health technologies might influence data interpretation. Analytical decisions and emerging interpretations were therefore regularly reviewed and challenged within the research team to enhance reflexivity and analytical rigor.

### Ethical Considerations

The study adhered to the general ethical principles of good clinical research practice as outlined in the World Medical Association’s Declaration of Helsinki [52]. All included interest-holders received written and oral information about the study, including its purpose, the voluntary nature of participation, the guarantee of anonymity, and the right to withdraw at any time. Written informed consent was obtained from all participants. Organization names and detailed role titles are not reported because the small number of national actors in some categories could permit deductive identification. Data storage and access complied with General Data Protection Regulation requirements, and personal data will be deleted at the end of the study or upon withdrawal of consent.

Under Danish law, only studies involving human biological material require approval from the National Committee on Health Research Ethics; observational studies do not require such approval [53]. Consequently, no formal ethical approval was required for this study, as confirmed by the Central Denmark Region Committees on Health Research Ethics (case number: 1-10-72-103-24).

## Results

### Overview

Across interest-holder groups and analytical framework levels, four overarching themes emerged, illustrating different tensions, competing values, and dilemmas: (1) improved access versus widening inequity, (2) efficiency versus relational and clinical quality, (3) digital transformation versus implementation uncertainty, and (4) strategic governance versus target-driven digitalization.

The access-equity tension was evident across all 4 interest-holder groups. Efficiency-quality concerns were particularly evident in accounts from professional interest and technology and digital health representatives, whereas transformation and governance concerns were most extensively elaborated by technology and digital health and public health authority representatives. Patient organization representatives particularly foregrounded equity, patient preferences, and potential safety concerns. These patterns indicate differences in emphasis rather than the prevalence of particular views within or between the groups.

### Theme 1: Improved Access Versus Widening Inequity

Across all 4 interest-holder groups, video consultations were perceived as having considerable potential to improve access to general practice. Participants highlighted reduced travel and time away from work as a particular advantage for working-age adults and follow-up consultations for care home residents and individuals with mobility limitations. A representative from a professional interest organization suggested that video consultations may be particularly useful for groups who might otherwise experience barriers to care or avoid seeking care:

Children who just need to be looked at in the early morning to see whether they are themselves ... that is typically just flexible and works well. I also think anxious and depressed patients. It has previously been shown in psychiatry that you can meet these groups in a different way. Those who did not dare to go outside their door ... suddenly you might be able to reach them. It may be that they will attend these consultations.

P12, professional interest organization

However, these perceived gains in accessibility were frequently accompanied by concerns that the benefits of video consultations may accrue primarily to digitally confident and resourceful citizens. Several participants challenged the assumption that increased digital availability automatically improves access for all patient groups. A representative from a patient organization emphasized that digital services must be adapted not only to broad target groups but also to substantial variation in individual digital skills and preferences:

Beyond target group assessments, there is also an individual assessment, which can be difficult, because we tend to design services for many people at once. Two fifty-year-olds with roughly the same background do not necessarily have the same digital skills.

P7, patient organization

Similarly, a representative from a professional interest organization questioned whether video consultations risk becoming primarily a service improvement for patients who are already capable of navigating digital health services, thereby reinforcing existing inequalities in access:

It is all very well that there is political enthusiasm for video, but now we are really just giving video to those who can already manage it ... it becomes a service improvement for those who are skilled, resourceful, and able to use such solutions.

P13, professional interest organization

Taken together, participants broadly agreed that video consultations may improve access and convenience for many patients. However, substantial concerns remained regarding whether these benefits would be distributed equitably across patient groups and whether digital expansion could unintentionally exacerbate existing inequalities.

### Theme 2: Efficiency Versus Relational and Clinical Quality

Some participants, particularly from professional interest organizations and technology and digital health organizations, described video consultations as enabling more focused, structured, and time-efficient interactions. One representative from a technology and digital health organization argued that video consultations can help align expectations between patients and clinicians by encouraging consultations to remain focused on the issue initially presented:

Instead of patients coming to their general practitioner with several additional issues that there is no time set aside for, video seems to align everyone around the time available ... this was the time, full stop. The eight minutes have been used, thank you very much. It becomes more concrete what the issue was, and that was it.

P14, technology and digital health organization

At the same time, many participants questioned whether greater focus and efficiency necessarily translated into better clinical encounters. Across several interest-holder groups, concerns were raised that video consultations may reduce opportunities to observe subtle but clinically relevant cues, limit the development of trust and rapport, and constrain the broader narrative processes through which patients communicate concerns. A representative from a technology and digital health organization noted that in-person encounters provide access to a wider range of sensory and contextual information, while also acknowledging that video consultations may offer unique insights into patients’ home environments:

There may be some things you cannot notice on video. It could be smells. It could be things like whether the patient looks dishevelled. There are simply more parameters you can assess in person, but on the other hand it is an advantage to see the patient and how they live at home.

P1, technology and digital health organization

Participants also expressed concerns that video consultations may weaken relational continuity and reduce clinicians’ confidence in their ability to assess the whole patient. According to one representative from a technology and digital health organization, some clinicians perceive video consultations as creating distance from patients and diminishing aspects of practice that originally motivated them to become doctors:

I actually think that what some of them [general practitioners] experience is that they feel they are losing contact with their patients ... part of what they became doctors for.

P14, technology and digital health organization

These concerns extended beyond clinical quality to questions of workload and efficiency. A representative from a professional interest organization highlighted the risk that video consultations may lead to duplication of work when patients subsequently require face-to-face assessment, thereby increasing workload for both clinicians and patients:

I think part of the concern is the risk of doing things twice. If I see the patient physically, I can complete the consultation. But if we start with a video consultation and I then have to say: you actually need to come in, then I have made things more expensive and wasted more time ... both my own and the patient's. In a busy day, that can feel like double work for the general practitioner.

P13, professional interest organization

Overall, participants differed in how they assessed the value of greater efficiency and focus. For some, video consultations represented a useful tool for streamlining some consultations. For others, the same characteristics raised concerns about reduced relational depth, missed information, and limitations in holistic clinical assessment.

### Theme 3: Digital Transformation Versus Implementation Uncertainty

Throughout the interviews, participants reflected on video consultations as part of a broader process of digital transformation in health care. A representative from a professional interest organization suggested that health care professionals should actively engage with this development to help ensure that digital solutions contribute positively to a coherent health care system:

It [implementation of video consultations] is probably about playing along with the healthcare system as a whole. It is about not digging your heels in and saying you don't want to be part of a development that we know will take place everywhere. From where I sit, as long as we can contribute to ensuring that general practitioners ... for the sake of citizens ... go along with this development as part of a coherent healthcare system, then I think we are well on our way.

P12, professional interest organization

Participants described video consultations as a means of overcoming organizational boundaries and facilitating collaboration between general practice, hospitals, and municipal services. A representative from a public health authority described opportunities to use video consultations to strengthen coordination across sectors, although noting that such solutions have not yet become widespread in routine practice:

I have heard that some hospitals can organise discharge conferences where you meet with the hospital, perhaps the municipality, and the general practitioner. These are exactly the kinds of integrations that video makes possible, but it is not something we have focused on very much. I have not noticed a particularly strong demand for it, which may also suggest that things are functioning reasonably well as they are.

P3, public health authority

However, a representative from a technology and digital health organization emphasized that successful cross-sector implementation remains challenging because it requires coordination across organizations with different cultures, workflows, and responsibilities:

I still see cross-sector video consultations as the more difficult part to implement, because it cuts across sectors and there are many different organisations that need to talk to each other...and also need some understanding of each other's cultures and workflows for it to run smoothly. Who takes responsibility, and things like that...that, I think, is still the hardest part.

P10, technology and digital health organization

At the same time, considerable uncertainty surrounded the practical implementation of these ambitions. A representative from a technology and digital health organization observed substantial variation in views regarding when video consultations are clinically appropriate, arguing that decisions about their use often depend on individual professional judgment and the specific clinical context:

I have been involved in many evaluations of video projects, and it varies a lot what people say video can be used for. Some say that if it is a wound, then you definitely cannot use video. Others say that if it is patients with dementia, then you also cannot use video. And others say you actually can use it for both. So I think it is very much up to the individual user to assess: can we use video in this situation? If the doctor is going to do a video consultation, they must evaluate: can we use this?

P10, technology and digital health organization

Taken together, participants viewed video consultations as part of a broader digital transformation with substantial potential to improve integration, coordination, and innovation. However, these opportunities were accompanied by uncertainty regarding implementation and the role of video consultations within everyday general practice.

### Theme 4: Strategic Governance Versus Target-Driven Digitalization

Participants also identified opportunities to strengthen implementation through reimbursement mechanisms, governance arrangements, and continuous technological development. A representative from a professional interest organization emphasized the importance of developing reliable and trustworthy digital tools that align with clinical workflows and support confident use in everyday practice:

Whether it is video or something else: tools have to be as good as we can make them. That is why we constantly have an interest in developing video solutions, so that they reflect workflows and needs, and have the operational stability that makes them trustworthy ... so that you can confidently use them with patients in general practice.

P13, professional interest organization

However, alongside these opportunities, participants repeatedly expressed concerns that video consultations risk becoming a policy objective. A representative from a public health authority cautioned that they should not become a goal in themselves:

It must not become an end in itself that we should have video, or more video.

P3, public health authority

Several participants also argued that metrics such as use rates could obscure more fundamental questions. A representative from a patient organization argued that patient satisfaction alone is an insufficient measure of quality and safety:

I very often hear the argument: “Well, the citizens are satisfied”. And I think that that's all very well, but they don't actually know what care they should be receiving. There has to be a professional judgement. It's a bit like saying: “Well, I was happy but I died”. That simply does not work.

P7, Patient Organization

A representative from a technology and digital health organization further argued that insufficient attention has been paid to rethinking how video consultations can be used constructively within general practice:

Nobody has really made the effort to figure out how video should be used constructively and well in general practice as such [...] nobody really took a step back and looked at video as a whole in a general practice context. What is it actually? What can we use it for? Could it be done differently? Instead, it is a bit like just adding electricity to something, rather than thinking whether it could be done in another way.

P9, technology and digital health organization

Participants also described concerns about implementation approaches perceived as overly top-down or politically driven. A representative from a technology and digital health organization suggested that successful implementation depends more on professional engagement and local champions than on external pressure or mandates:

One of the driving forces behind it was political. At the beginning, it felt more like a stick ... now you must do video ... rather than a carrot. And my point is that if you want to create change, it may be better to spark interest in why something is actually good, and to find ambassadors among general practitioners who can speak up for it, rather than standing with a stick and thinking you can force it through.

P14, technology and digital health organization

Overall, these findings suggest that interest-holders largely supported the continued development of video consultations but differed in their views regarding how implementation should be governed. While participants recognized the importance of supportive infrastructures, incentives, and national coordination, they also warned against approaches that prioritize technological uptake, activity targets, or political ambitions over clinical value, quality, and patient safety.

## Discussion

### Main Findings

This study explored organizational interest-holder perspectives on the implementation of video consultations in Danish general practice. The findings were characterized by 4 recurring tensions, competing values, and dilemmas: improved access versus widening inequity; efficiency versus relational and clinical quality; digital transformation versus implementation uncertainty; and strategic governance versus target-driven digitalization. Participants broadly agreed that video consultations may improve accessibility and convenience but questioned whether these benefits would be distributed equitably. Similarly, perceived efficiency gains were balanced against concerns about relational continuity and potential patient safety risks. Video consultations were viewed as part of a broader digital transformation offering opportunities for integration and coordination across health care sectors. However, uncertainty remained regarding implementation and routine use in general practice. Finally, participants highlighted concerns that video consultations risk becoming policy objectives in themselves. While supportive infrastructures and incentives were considered important, participants emphasized that implementation should be guided by clinical value, quality of care, and patient safety rather than use targets alone. Taken together, the findings suggest that implementing video consultations is not simply a technological challenge but a process of balancing competing priorities and values across health care organizations, professions, and systems.

### Comparison With Previous Literature

The principal contribution of this study lies less in identifying tensions between access and equity or between efficiency and quality, both of which are established in digital health research, than in showing how organizational representatives may assign different values to the same feature of video consultation. These differing assessments reflect their respective mandates, responsibilities, and understandings of good care and governance. Video consultation should therefore not be understood as having a fixed organizational or clinical value: what constitutes an advantage for one interest-holder may represent a burden, risk, or loss of quality for another.

Previous research has shown that video consultations may reduce travel and time burdens, lower thresholds for contact, and increase flexibility and continuity for selected patient groups [10,12,13,25,29,30,54]. Representatives across all 4 interest-holder groups recognized these possibilities but rarely regarded improved access as an unqualified benefit. Greater convenience for digitally confident patients could coexist with new barriers for patients with limited digital resources, unsuitable clinical needs, or a preference for nondigital care [12,30,38]. Similarly, the more focused interactions associated with video consultations could be interpreted as increasing efficiency while also narrowing opportunities to identify additional concerns, observe nonverbal or physical cues, establish rapport, or complete an assessment without a subsequent face-to-face consultation [10,25,29,55-59]. Access, efficiency, equity, and quality were therefore not described as independent outcomes but as competing definitions of value. Their relative importance depended on which patients were considered, which consequences were prioritized, and whether quality was understood in terms of convenience, throughput, relational continuity, clinical completeness, safety, or equitable access.

A similar pattern was evident at organizational and system levels. Video consultations have previously been described as components of broader digital transformation, with potential to support integrated care pathways, cross-sector collaboration, and service redesign [22,29-31,60,61]. However, these opportunities depended on interoperable infrastructure, reliable technologies, workable divisions of responsibility, and alignment with clinical workflows. Representatives also highlighted how benefits and burdens could be distributed unevenly: a solution that supported system-level ambitions for scaling or coordination could create additional work, uncertainty, or costs within another organization or at the point of care. Concerns about reimbursement, performance targets, accountability, and cross-sector cost shifting consequently concerned not only implementation barriers but also who was able to define successful implementation and whose costs and outcomes were included in that assessment [29-31,39,62,63].

This organizational lens extends patient- and clinician-focused research by showing that implementation involves negotiation between competing definitions of value across the health care system [26-28]. Organizational representatives from the 4 competing interest-holder groups approached video consultation from different positions and forms of responsibility, shaping what they regarded as appropriate use, acceptable risk, relevant evidence, and successful implementation. Governance and implementation strategies must therefore ask not only whether video consultations increase access or efficiency, but when they add value, for whom, under what conditions, at what cost, and according to whose understanding of quality. Increasing uptake without making these assumptions explicit risks privileging system-level or technological objectives over clinical appropriateness, relational quality, professional autonomy, or equity [64].

### Strengths and Limitations

To our knowledge, this study is among the first to explore organizational interest-holder perspectives on the implementation of video consultations in general practice through a qualitative analysis. A key strength of the study is the inclusion of 4 distinct interest-holder groups: patient organizations, professional interest organizations, technology and digital health organizations, and public health authorities. Participants occupied senior or specialist positions and therefore contributed perspectives grounded in governance, policy development, clinical practice, advocacy, and technological implementation. This enabled examination of implementation from multiple organizational vantage points rather than from a single professional or user perspective. Another strength is the study’s analytical focus on tensions and competing interpretations of implementation. Rather than identifying only barriers and facilitators, the analysis investigates how the same feature of video consultations may be interpreted differently depending on organizational responsibilities, priorities, and forms of expertise. This provides a more nuanced understanding of implementation as a process of negotiating competing values, including access and equity, efficiency and quality, and top-down governance and professional autonomy.

Several limitations should also be acknowledged. First, the study was conducted within a digitally mature, high-income health care system with extensive experience of digital health technologies. While many of the identified themes are likely relevant beyond Denmark, transferability to other health care systems should be considered cautiously. Second, purposive and snowball sampling may have favored organizations with a particular interest in digital health, potentially influencing the issues emphasized during interviews. Third, although the study included a diverse range of organizations, the interest-holder groups were uneven in size, with more participants representing public health authorities (n=7) than patient organizations (n=3), professional interest organizations (n=3), or technology and digital health organizations (n=4). Consequently, governance and system-coordination concerns may have been more extensively elaborated than patient- and profession-specific concerns. The prominence of a theme should therefore not be interpreted as its prevalence across the wider organizational landscape, and the findings should be understood as analytically informative perspectives rather than representative views of all actors within each category. Finally, the study focuses on organizational perspectives rather than direct experiences of patients or health care professionals. This was consistent with the study aim but means that the findings primarily illuminate how implementation is understood and governed at organizational and system levels. Despite these limitations, the study contributes new insights into the competing priorities and implementation logics that shape the adoption of video consultations in general practice.

### Implications for Research, Practice, and Policy

The findings suggest that future research should move beyond questions of whether video consultations work and focus more explicitly on how and why they add value in general practice, with particular attention to the 4 tensions identified in this study and how they influence their use in everyday general practice. Such research would contribute to a more nuanced understanding of implementation as a complex, dynamic sociotechnical and organizational process rather than a simple matter of technology adoption [48,64-67].

For clinical practice, the findings highlight the importance of moving beyond a binary debate about increasing or reducing video consultation use. Instead, greater attention should be directed toward identifying appropriate use cases and supporting clinicians in making context-sensitive decisions about when video consultations add value [24,68-70]. Education and training should therefore include virtual communication, remote clinical assessment, and risk management to support safe and effective use of video consultations [71-73].

At policy and system levels, the findings suggest that successful implementation may depend on balancing competing values rather than simply promoting the widespread use of video consultations [20,21]. This includes aligning incentives, reimbursement models, and governance frameworks with clinical appropriateness, ensuring equitable access for patients with varying levels of digital literacy, vulnerability, and care preferences, and investing in interoperable digital infrastructure and cross-sector collaboration to support coherent implementation and long-term integration across health care sectors [64,74-76].

More broadly, the findings highlight the importance of sustained dialogue between policymakers, clinicians, patient representatives, and technology providers [22,49,77]. The implementation of video consultations is not solely a technical challenge but a process of negotiating different understandings of value, quality, safety, and success. Governance approaches that recognize and actively engage with these differing perspectives may therefore be better positioned to support the long-term integration of video consultations into high-quality general practice.

## Appendix

Multimedia Appendix 1 Topic guide.

Multimedia Appendix 2 Analytical process from initial framework analysis to higher-order interpretative themes.

## Abbreviations

CIOMS: Council for International Organizations of Medical Sciences
COREQ: Consolidated Criteria for Reporting Qualitative Research
SWOT: Strengths, Weaknesses, Opportunities, and Threats
