# Supplementary material for: Strategic Considerations for Video Consultation Implementation in General Practice: Qualitative Study of Organizational Interest-Holders’ Perspectives
Source: J Med Internet Res. 2026 Oct 19;28:e106707. doi: 10.2196/106707 (PMC13728767; doi:10.2196/106707)
Supplement: Supplementary file 1 [file jmir_v28i1e106707_app1.docx]

**Multimedia Appendix 1: Topic guide**

***PART I: Individual experiences***

- Could you begin by telling us about your background and your role in the (company/organisation)?
- How long have you worked at the (company/organisation)?
- Who is the (company/organisation), and what role do you play in relation to the use of video consultations within the healthcare system?

***PART II: SWOT***

- What advantages/strengths do you see in video consultations?
- What works particularly well?
- What disadvantages/weaknesses do you see in video consultations?
- What works less well?
- What opportunities do video consultations offer, and what potentials do they hold?
- What barriers and threats do you experience in relation to the development and implementation of video consultations?
- How do you address them?
- What are your expectations or ambitions regarding the future implementation of video consultations?
- What are you working towards?

***Closing***

- Is there anything else you would like to add?
