# Supplementary material for: Strategic Considerations for Video Consultation Implementation in General Practice: Qualitative Study of Organizational Interest-Holders’ Perspectives
Source: J Med Internet Res. 2026 Oct 19;28:e106707. doi: 10.2196/106707 (PMC13728767; doi:10.2196/106707)
Supplement: Supplementary file 2 [file jmir_v28i1e106707_app2.docx]

**Multimedia Appendix 2. Analytical process from initial framework analysis to higher-order interpretative themes**

This appendix presents the analytical process used to move from the initial framework analysis to the development of
higher-order interpretative themes. The analysis was conducted in two stages:

1. Data were analysed within the five predefined analytical framework levels (patients, healthcare organisation units,
   healthcare systems, legislation and regulation, and technology and infrastructure), generating descriptive findings
   summarised in **Tables S1–S5**.
2. Patterns across the five analytical framework levels were then compared and synthesised through an interpretative
   analysis to identify broader themes that captured common tensions, competing values, and dilemmas associated
   with video consultations in general practice. The resulting four higher-order themes therefore represent a synthesis
   of the perspectives of interest-holder organisations on video consultations in general practice rather than findings
   confined to a single analytical level.

**Table S1.** Patients' perceived strengths, weaknesses, opportunities, and threats across interest-holder groups**.**

| **Interest-holder group** | **Strengths** | **Weaknesses** | **Opportunities** | **Threats** |
| --- | --- | --- | --- | --- |
| *Patient  organisations* | Improved accessibility and flexibility | Risk of missed clinical cues and reduced reassurance | Expanded access and lower thresholds for care | Widening digital inequities |
|  | Reduced travel and time burden | Loss of embodied clinical assessment | Inclusion of underserved or mobility-limited groups | Reduced safety for vulnerable patient groups |
|  | Comfort and safety of consultations from home | Uneven digital competence among patients | Integration with home monitoring technologies |  |
| *Professional interest  organisations* | Improved accessibility and flexibility | Risk of missed clinical cues and reduced reassurance | Expanded access and lower thresholds for care | Widening digital inequities |
|  | Lower threshold for contacting general practice | Loss of embodied clinical assessment | Inclusion of underserved or mobility-limited groups | Reduced safety for vulnerable patient groups |
|  |  | Limited experience with video consultations |  | Misalignment with patient preferences |
|  |  |  |  | Risk that resources shift away from vulnerable patients |
| *Technology and digital health organisations* | Improved accessibility and flexibility | Risk of missed clinical cues and reduced reassurance | Expanded access and lower thresholds for care |  |
|  | Reduced travel and time burden | Loss of embodied clinical assessment | Inclusion of underserved or mobility-limited groups |  |
|  | Comfort and safety of consultations from home | Uneven digital competence among patients | Integration with home monitoring technologies |  |
|  | Lower threshold for contacting general practice | Limited experience with video consultations | Possibility for relatives to participate in consultations |  |
| *Public  health  authorities* | Improved accessibility and flexibility |  | Expanded access and lower thresholds for care | Widening digital inequities |
|  |  |  | Inclusion of underserved or mobility-limited groups |  |
|  |  |  | Possibility for relatives to participate in consultations |  |

**Table S2.** Healthcare organisation units' perceived strengths, weaknesses, opportunities, and threats across interest-holder groups.

| **Interest-holder group** | **Strengths** | **Weaknesses** | **Opportunities** | **Threats** |
| --- | --- | --- | --- | --- |
| *Patient  organisations* |  | Professional uncertainty and lack of guidelines |  | Risk of missing important clinical information |
|  |  |  |  | Pressure to adopt technologies without sufficient evidence |
| *Professional interest  organisations* | More focused and efficient consultations | Loss of embodied clinical contact | New ways of organising work in general practice | Risk of missing important clinical information |
|  | Alignment between  patient expectations and  GP assessment | Professional uncertainty and lack of guidelines | Supporting professional sustainability | Erosion of relational continuity in care |
|  | Existing GP-patient relationships support video use | Limited training in virtual communication | Development of GPs as digital ambassadors | Time displacement and potential workload increase |
|  |  | Limited experience with video consultations |  | Pressure to adopt technologies without sufficient evidence |
| *Technology and digital health organisations* | More focused and efficient consultations | Loss of embodied clinical contact | New ways of organising work in general practice | Risk of missing important clinical information |
|  | Alignment between  patient expectations and  GP assessment | Professional uncertainty and lack of guidelines | Development of GPs as digital ambassadors | Erosion of relational continuity in care |
|  | Insight into patients’ home environments | Limited experience with video consultations | Integration with home monitoring technologies | Pressure to adopt technologies without sufficient evidence |
| *Public  health  authorities* |  | Professional uncertainty and lack of guidelines |  |  |
|  |  |  |  |  |
|  |  |  |  |  |

**Table S3.** Healthcare systems' perceived strengths, weaknesses, opportunities, and threats across interest-holder groups.

| **Interest-holder group** | **Strengths** | **Weaknesses** | **Opportunities** | **Threats** |
| --- | --- | --- | --- | --- |
| *Patient  organisations* | Increased reach across geographical distances | Lack of a robust evidence  base | Integration of digital services and artificial intelligence | Fragmented implementation |
|  | Efficiency gains and improved capacity utilisation | Organisational uncertainty about appropriate use |  | Misaligned incentives |
|  | Potential to reduce  distance barriers |  |  | Worsening health inequalities |
| *Professional interest  organisations* | Reduced infection risks  in waiting rooms | Limited evaluation following rapid implementation |  | Fragmented implementation |
|  |  | Lack of a robust evidence  base |  | Misaligned incentives |
|  |  | Uneven use across  providers |  | Worsening health inequalities |
|  |  | Organisational uncertainty about appropriate use |  |  |
| *Technology and digital health organisations* | Increased reach across geographical distances | Limited evaluation following rapid implementation | Improved coordination and cross-sector collaboration | Fragmented implementation |
|  | Efficiency gains and improved capacity utilisation | Uneven use across  providers | Integration of digital services and artificial intelligence | Misaligned incentives |
|  | Potential to reduce  distance barriers | Organisational uncertainty about appropriate use | More integrated care pathways | Cross-sector cost shifting |
|  |  |  | Standardised national  digital services |  |
| *Public  health  authorities* | Increased reach across geographical distances | Organisational uncertainty about appropriate use | Improved coordination and cross-sector collaboration | Fragmented implementation |
|  |  |  | More integrated care pathways | Misaligned incentives |
|  |  |  | Standardised national  digital services | Cross-sector cost shifting |

**Table S4.** Legislation and regulations' perceived strengths, weaknesses, opportunities, and threats across interest-holder groups.

| **Interest-holder group** | **Strengths** | **Weaknesses** | **Opportunities** | **Threats** |
| --- | --- | --- | --- | --- |
| *Patient  organisations* |  | Ambiguity around responsibility and  safety threshold |  | Target-driven digitalisation |
|  |  |  |  | Volume over value |
|  |  |  |  | Excessive focus on economic incentives |
| *Professional interest  organisations* |  | Lack of consistent frameworks across sectors |  | Target-driven digitalisation |
|  |  |  |  | Volume over value |
|  |  |  |  |  |
| *Technology and digital health organisations* | Strong national digital  health infrastructure | Ambiguity around responsibility and  safety threshold | Alignment of incentives  for digital care | Target-driven digitalisation |
|  |  | Lack of consistent frameworks across sectors | National scaling of video consultations |  |
|  |  | Unclear cross-sector accountability | Shared governance across sectors |  |
|  |  |  |  |  |
| *Public  health  authorities* | Strong national digital  health infrastructure | Ambiguity around responsibility and  safety threshold | Alignment of incentives  for digital care | Target-driven digitalisation |
|  | Public digital services  supporting access | Lack of consistent frameworks across sectors | National scaling of video consultations | Volume over value |
|  | Regulatory support for  digital solutions | Unclear cross-sector accountability | Shared governance across sectors | Excessive focus on economic incentives |

**Table S5.** Technology and infrastructures' perceived strengths, weaknesses, opportunities, and threats across interest-holder groups.

| **Interest-holder group** | **Strengths** | **Weaknesses** | **Opportunities** | **Threats** |
| --- | --- | --- | --- | --- |
| *Patient  organisations* |  | Uneven user competence |  |  |
|  |  |  |  |  |
|  |  |  |  |  |
| *Professional interest  organisations* |  |  | Broader adoption across sectors | Difficulties implementing cross-sector solutions |
|  |  |  |  |  |
|  |  |  |  |  |
| *Technology and digital health organisations* | Mature digital infrastructure | Technical instability | Scalable digital ecosystems | Vendor dependency |
|  | User-friendly mobile solutions | Uneven bandwidth and connectivity | Reuse of successful technological solutions | Outdated legacy systems |
|  | Existing national platforms | Uneven user competence | Gradual expansion of digital services | Persistent technological siloing |
|  | Strong vendor expertise | Limited interoperability | Broader adoption across sectors | Difficulties implementing cross-sector solutions |
| *Public  health  authorities* | Mature digital infrastructure |  | Scalable digital ecosystems | Persistent technological siloing |
|  | User-friendly mobile solutions |  | Gradual expansion of digital services | Difficulties implementing cross-sector solutions |
|  | Existing national platforms |  | Broader adoption across sectors |  |

**Development of Higher-Order Interpretative Themes**

To move beyond descriptive categorisation, findings from all five analytical framework levels were compared iteratively.

Areas of convergence, divergence, tension, and overlap were identified across organisational interest-holder groups.

This process resulted in four higher-order interpretative themes:

1) Improved access versus widening inequity.
2) Efficiency versus relational and clinical quality.
3) Digital transformation versus implementation uncertainty.
4) Strategic governance versus target-driven digitalisation.

**Theme 1: Improved access versus widening inequity**

This comes primarily from **Supplementary Table 1**.

The *access* side includes:

- Improved accessibility and flexibility
- Reduced travel and time burden
- Lower threshold for contacting general practice
- Expanded access and lower thresholds for care
- Inclusion of underserved or mobility-limited groups

The *equity* side includes:

- Widening digital inequities
- Reduced safety for vulnerable patient groups
- Misalignment with patient preferences
- Risk that resources shift away from vulnerable patients
- Uneven digital competence among patients

The analytical insight is that the same development that improves access for some patients may simultaneously create barriers for others.

**Theme 2: Efficiency versus relational and clinical quality**

This emerges mainly from **Supplementary Table 2**.

The *efficiency* side includes:

- More focused and efficient consultations
- Alignment between patient expectations and GP assessment
- New ways of organising work in general practice
- Supporting professional sustainability

The *quality* side includes:

- Loss of embodied clinical contact
- Risk of missing important clinical information
- Erosion of relational continuity
- Professional uncertainty
- Limited training in virtual communication
- Time displacement and potential workload increase

The analytical insight is that efficiency and focus are not universally regarded as improvements;
they may come at the expense of clinical and relational qualities valued in general practice.

**Theme 3: Digital transformation versus implementation uncertainty**

This emerges from **Supplementary Tables 3** and **5** .

The *transformation* side includes:

- Improved coordination and cross-sector collaboration
- More integrated care pathways
- Standardised national services
- Integration of AI
- Scalable digital ecosystems
- Broader adoption across sectors

The *uncertainty* side includes:

- Organisational uncertainty about appropriate use
- Limited evaluation following rapid implementation
- Technical instability
- Limited interoperability
- Difficulties implementing cross-sector solutions
- Fragmented implementation

The analytical insight is that participants support digital transformation but remain uncertain about how it should be implemented in practice.

**Theme 4: Strategic governance versus target-driven digitalisation**

This emerges most clearly from **Supplementary Tables 3** and **4**.

The *strategic governance* side includes:

- Strong national digital infrastructure
- Public digital services supporting access
- Alignment of incentives
- Shared governance
- National scaling

The *target-driven digitalisation* side includes:

- Target-driven digitalisation
- Volume over value
- Excessive focus on economic incentives
- Misaligned incentives
- Cross-sector cost shifting

The analytical insight is that participants generally support governance and coordination
but oppose implementation approaches that prioritise uptake metrics over clinical value.
